# Intracellular trafficking of TREM2 is regulated by presenilin 1

**DOI:** 10.1038/emm.2017.200

**Published:** 2017-12-01

**Authors:** Yingjun Zhao, Xiaoguang Li, Timothy Huang, Lu-lin Jiang, Zhenqiu Tan, Muxian Zhang, Irene Han-Juo Cheng, Xin Wang, Guojun Bu, Yun-wu Zhang, Qi Wang, Huaxi Xu

**Affiliations:** 1 Neuroscience Initiative, Sanford Burnham Prebys Medical Discovery Institute, La Jolla, CA, USA; 2 Fujian Provincial Key Laboratory of Neurodegenerative Disease and Aging Research, Institute of Neuroscience, The Collaborative Innovation Center for Brain Science, Medical College, Xiamen University, Xiamen, China; 3 Institute of Clinical Pharmacology, Guangzhou University of Chinese Medicine, Guangzhou, China; 4 Institute of Brain Science, National Yang-Ming University, Taipei, Taiwan

## Abstract

Genetic mutations in triggering receptor expressed on myeloid cells 2 (TREM2) have been linked to a variety of neurodegenerative diseases including Alzheimer’s disease, amyotrophic lateral sclerosis, frontotemporal dementia and Parkinson’s disease. In the brain, TREM2 is highly expressed on the cell surface of microglia, where it can transduce signals to regulate microglial functions such as phagocytosis. To date, mechanisms underlying intracellular trafficking of TREM2 remain elusive. Mutations in the presenilin 1 (PS1) catalytic subunit of the γ-secretase complex have been associated with increased generation of the amyloidogenic Aβ (amyloid-β) 42 peptide through cleavage of the Aβ precursor amyloid precursor protein. Here we found that TREM2 interacts with PS1 in a manner independent of γ-secretase activity. Mutations in TREM2 alter its subcellular localization and affects its interaction with PS1. Upregulation of PS1 reduces, whereas downregulation of PS1 increases, steady-state levels of cell surface TREM2. Furthermore, PS1 overexpression results in attenuated phagocytic uptake of Aβ by microglia, which is reversed by TREM2 overexpression. Our data indicate a novel role for PS1 in regulating TREM2 intracellular trafficking and pathophysiological function.

## Introduction

Recently, several heterozygous missense mutations in triggering receptor expressed on myeloid cells 2 (TREM2) have been identified as risk factors for a number of neurodegenerative disorders including Alzheimer’s disease (AD), amyotrophic lateral sclerosis, frontotemporal dementia and Parkinson’s disease.^1, 2, 3, 4, 5, 6, 7, 8, 9, 10, 11, 12^ For example, the *TREM2 R47H* variant has been reported to confer an increased risk of AD in comparable odds ratios to *APOE4*,^8, 9^ the greatest known genetic risk factor for late-onset AD.^13^

**Figure 1:**
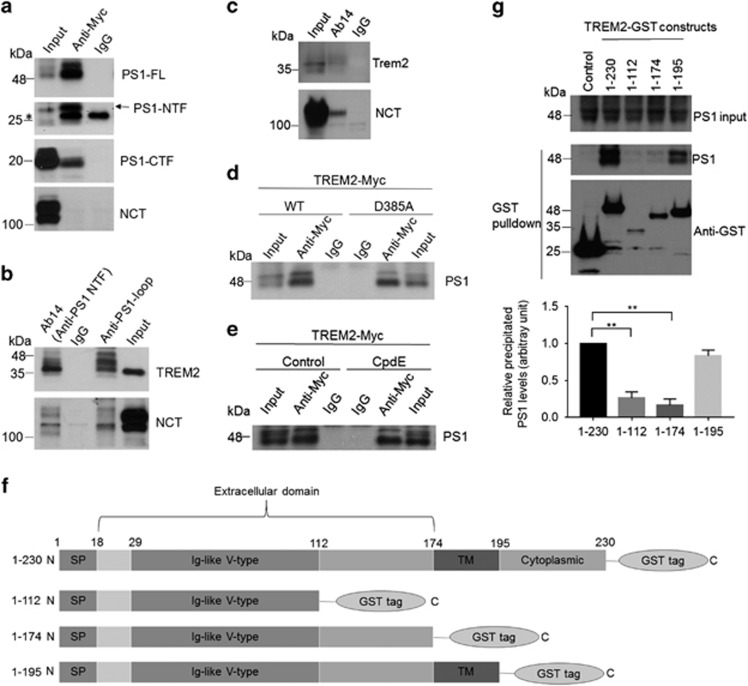
Presenilin 1 (PS1) interacts with TREM2. (**a**, **b**) PS1 constructs were transfected into HEK293 cells stably expressing TREM2 with a Myc-tag at the C terminus (HEK293-TREM2). (**a**) Cell lysates were immunoprecipitated with a Myc antibody or control IgG. Immunoprecipitated proteins were subjected to immunoblotting with an Ab14 antibody to detect full-length PS1 (PS1-FL) and the PS1 N-terminal fragment (NTF), an anti-PS1 loop antibody to detect the PS1 C-terminal fragment (CTF) and a nicastrin (NCT) antibody as indicated. (**b**) Cell lysates were immunoprecipitated with Ab14, anti-PS1 loop or control IgG and immunoblotted with Myc and NCT antibodies. (**c**) Lysates from BV2 microglial cells were immunoprecipitated with Ab14 and immunoblotted with NCT and mouse TREM2 antibodies. (**d**) Vectors expressing wild-type (WT) or mutant PS1 (D385A) were transfected into HEK293-TREM2 cells. Cell lysates were immunoprecipitated with Ab14 or control IgG, and PS1 was detected by immunoblotting. (**e**) Lysates from HEK293-TREM2 cells with or without Compound E (CpdE, a γ-secretase inhibitor) treatment were immunoprecipitated with Ab14 or control IgG, and PS1 was detected by immunoblotting. (**f**) Schematic representations of full-length (1–230) or truncated TREM2 constructs, all tagged with GST at the C terminus. SP, signal peptide; TM, transmembrane domain. (**g**) PS1 was co-expressed with full-length TREM2 or other TREM2 fragments as shown in **f** in HEK293 cells. Cell lysates were precipitated with Glutathione Sepharose beads and immunoblotted with the PS1 antibody Ab14 or an antibody against GST. PS1 co-precipitation levels were determined by densitometric analysis and normalized with respect to both PS1 expression and precipitated GST. **P<0.01, *n*=3, Student’s *t*-test.

TREM2 is a type I transmembrane protein comprising an extracellular, Ig-like V-type domain; a transmembrane domain; and an intracellular domain lacking any obvious signaling motifs (Figure 1f). Signal transduction induced by ligand engagement, for example, ApoE,^14, 15, 16^ with the TREM2 extracellular domain is mediated through its association with DNAX-activating protein of 12 kDa (DAP12), which triggers intracellular signals through an immunoreceptor tyrosine-based activation motif.^17, 18^ TREM2 expression is primarily found in microglia, and mounting evidence indicates that TREM2 signaling is important in the regulation of microglial phagocytosis and inflammatory cytokine production.^19, 20, 21, 22, 23, 24, 25, 26, 27, 28, 29, 30^ Several disease-associated TREM2 mutations have been reported to reduce cell surface TREM2 distribution and impair microglial phagocytic function.^31, 32^ Therefore, the presence of TREM2 at the cell surface is important in mediating microglial functions such as phagocytosis. However, the precise mechanism by which TREM2 is distributed to the cell surface or intracellular compartments remains largely unknown.

Interestingly, it has been reported that TREM2 can be cleaved by the γ-secretase complex,^31, 33^ which is required for the generation of amyloid-β (Aβ) peptides that comprise pathological senile AD plaques.^34^ Aberrant Aβ accumulation can trigger a cascade of neurodegenerative events including synaptic dysfunction, oxidative injury and eventual neuronal loss.^35, 36^ Importantly, microglia-mediated phagocytosis has been suggested to be involved in clearing extracellular Aβ deposits in the brain.^37^ The γ-secretase complex is composed of four primary components: presenilins (PSs, including PS1 and PS2), anterior pharynx-defective-1 (APH-1), nicastrin (NCT) and presenilin enhancer-2 (PEN-2).^38, 39^ PS1 is the major presenilin component in the catalytically active γ-secretase complex, where autoproteolytically derived amino-terminal fragments (NTFs) and carboxyl-terminal fragments (CTFs) form functional heterodimers.^40^ PEN-2 is known to cleave full-length PS1 to generate NTF and CTF constituents that form stable heterodimers. APH-1 is required for the assembly of premature components and for the proteolytic activity of the complex, and NCT plays a primary role in the intracellular trafficking and the stability of the assembled complex.^41^ Numerous PS1 mutations have been identified in early-onset familial AD patients.^42^ Although PS1 is believed to be the catalytic subunit of γ-secretase, additional functions have been proposed for PS1, including the regulation of vesicular trafficking,^43, 44^ calcium homeostasis,^45, 46^ autophagy^47, 48^ and other cellular functions.^49^

Here we show that PS1 can interact with TREM2 to affect TREM2-mediated phagocytic capacity in microglia. Our results identify a novel mechanism underlying the regulation of intracellular TREM2 trafficking and provide insight into functional interactions between two fundamental AD risk components.

## Materials and methods

### Antibodies and reagents

Sulfo-NHS-LC-biotin, streptavidin agarose resin beads, Dynabeads Protein G, Turbofect Transfection Reagent and restriction enzymes were purchased from Thermo Fisher Scientific (Carlsbad, CA, USA). Glutathione Sepharose 4B was purchased from GE Healthcare Life Sciences (Piscataway, PA, USA). The γ-secretase inhibitor Compound E was purchased from Millipore (San Diego, CA, USA). 6-carboxyfluorescein (FAM)-Aβ was purchased from AnaSpec (Fremont, CA, USA). The following antibodies were used in this study: mouse anti-Myc (for immunoblot, Life Technologies, Carlsbad, CA, USA); rabbit anti-Myc (for immunostaining) and rabbit anti-PDI (Cell Signaling Technology, Danvers, MA, USA); sheep anti-Myc (for immunostaining, Thermo Fisher, Carlsbad, CA, USA); sheep anti-TGN46 (GeneTex, Irvine, CA, USA); mouse anti-nicastrin (Abcam, Cambridge, MA, USA); rabbit anti-PS1-NTF (Ab14) antibody (developed in-house previously); mouse anti-PS1 loop (Millipore); goat anti-human TREM2 and sheep anti-mouse TREM2 (R&D Systems, Minneapolis, MN, USA); mouse anti-β-actin (Sigma, St Louis, MO, USA); rabbit anti-GST, normal mouse IgG and rabbit IgG (Santa Cruz Biotechnology, Dallas, TX, USA).

### Cell culture and generation of stable cell lines

HEK293 cells and murine microglial BV2 cells were grown in Dulbecco’s modified Eagle’s medium (DMEM) (Corning, Corning, NY, USA) supplemented with 10% fetal bovine serum (HyClone, Piscataway, PA, USA). To generate HEK293 cells stably overexpressing human TREM2, we transfected cells with pcDNA3.1 vectors (Thermo Fisher) with Myc-tagged WT or mutated TREM2 cDNA inserts using Turbofect and selected for G418 resistance (400 μg ml^−1^). A lentiviral vector comprising human PS1 cDNA-IRES-mCherry was purchased from GeneCopoeia, Rockville, MD, USA and packaged into lentiviral particles (Lv-PS1) through the viral core at Sanford Burnham Prebys Medical Discovery Institute. For stable PS1 overexpression, BV2 cells were transduced with Lv-PS1, and individual mCherry-positive cells were sorted by flow cytometry using a high-speed cell sorter (BD Biosciences, San Jose, CA, USA, FACSAria IIu), after which single clonal cell lines were expanded and characterized for expression.

### RNA interference

Two PS1-targeting siRNAs (1, 5′-CCACACCATGTTGGAAATAAA-3′ 2, 5′-CCCACTTGTAAGTTTAAATAA-3′) and a scrambled control siRNA (Qiagen, Germantown, MD, USA) were transfected into BV2 cells using Lipofectamine RNAiMAX reagent (Invitrogen, Carlsbad, CA, USA) following the manufacturer’s protocol.

### Co-immunoprecipitation

HEK293 cells stably overexpressing WT or mutant TREM2 were transfected with or without pAG3 vectors for WT or D385A mutant PS1. Cells were lysed in lysis buffer (1% Nonidet P-40, 50 mM Tris-HCl, 150 mM sodium chloride, 2 mM EDTA, pH 7.4, supplemented with protease inhibitor mixture). Alternatively, BV2 microglia cells were lysed in CelLytic M buffer (Sigma) containing a protease inhibitor mixture. Lysates were incubated with normal IgG or the indicated antibodies together with Dynabeads Protein G at 4 °C overnight. Immunoprecipitated proteins were analyzed by immunoblotting.

### GST pulldown

HEK293 cells were transfected with PS1 and various GST-tagged TREM2 constructs (Figure 1e). Cells were lysed and incubated with Glutathione Sepharose beads at 4 °C overnight. Precipitated proteins were analyzed by immunoblotting.

### Cell surface biotinylation

Biotinylation was performed using a previously described protocol.^50^ Cells were washed three times with ice-cold PBS/CM (phosphate-buffered saline containing 1.3 mM CaCl_2_ and 1 mM MgCl_2_) and incubated with 0.5 mg ml^−1^ sulfo-NHS-LC-biotin for 20 min at 4 °C. Cell lysates were precipitated with streptavidin beads overnight, and the precipitated biotinylated proteins were subjected to immunoblot analysis.

### Immunoblot

Samples from co-IP, GST pulldown and cell surface biotinylation were subjected to sodium dodecyl sulfate-polyacrylamide gel electrophoresis and probed using the indicated antibodies.

### Immunostaining

HEK293 cells stably overexpressing WT or mutant TREM2 were transfected with PS1 expression constructs. Twenty-four hours after transfection, cells were fixed in 4% paraformaldehyde (PFA), permeabilized and blocked in 5% bovine serum albumin (BSA). The cells were then incubated with the indicated primary antibodies at 4 °C overnight. The cells were washed three times with PBS and then incubated with secondary antibodies conjugated to Alexa Fluor 488, 555 and 647 (Life Technologies). For cell surface labeling experiments, cells were blocked in 5% BSA after fixation and stained with a goat anti-human TREM2 antibody (epitope 19–174 amino acids, R&D Systems). The cells were then permeabilized and stained with a Myc antibody to detect total TREM2. Specimens were visualized using a confocal microscope (Zeiss, Oberkochen, Germany, LSM 710). Pearson’s correlation coefficient was determined by Zen software to quantify colocalization between TREM2 and TGN46/PDI, between PS1 and TGN46/PDI, and between TREM2 and PS1. BV2 cells were fixed in 4% PFA and subjected to processing for imaging as described above.

### Phagocytosis of FAM-Aβ

Treated BV2 microglial cells were seeded in 12-well plates at a density of 500 000 cells per well. After 24 h, the cells were incubated with media containing FAM-Aβ (500 nM) for 2 h. The cells were fixed with 4% PFA and visualized by confocal microscopy (Zeiss LSM 710). Alternatively, extracellular and cell surface FAM-Aβ was quenched by incubation with 0.4% trypan blue in PBS (pH 4.4) for 1 min. The cells were then trypsinized, washed with ice-cold PBS and subjected to flow cytometry analysis using an LSRFortessa X-20 cell analyzer (BD Biosciences).

### Statistical analyses

Statistical analyses were performed with GraphPad Prism (La Jolla, CA, USA). *N* represents the number of biological replications. Student’s *t*-test or one-way ANOVA was used. All data are presented as the mean±s.d.

## Results

To identify proteins that may regulate TREM2 trafficking, we generated a human embryonic kidney 293 cell line stably overexpressing Myc-tagged TREM2 (HEK293-TREM2) and performed co-immunoprecipitation (co-IP) assays to screen for TREM2-interacting components. Using this system, we identified positive interactions between TREM2 and PS1: with PS1 overexpression in HEK293-TREM2 cells, a Myc antibody immunoprecipitated full-length PS1, PS1-NTF and PS1-CTF, while no interaction between TREM2 and NCT was detected (Figure 1a). Conversely, TREM2 was co-precipitated using antibodies against PS1-NTF (Ab14) or PS1-CTF (anti-PS1 loop) (Figure 1b). Moreover, we found that endogenous TREM2 co-immunoprecipitated with endogenous PS1 in BV2 microglial cells (Figure 1c). The catalytically inactive dominant negative PS1 mutant (D385A)^51^ retained interaction with TREM2 comparable to wild-type (WT) PS1 (Figure 1d). Additionally, inhibition of γ-secretase activity with Compound E (CpdE) did not affect PS1/TREM2 co-precipitation (Figure 1e). These results demonstrate that PS1 interacts with TREM2 and that the interaction occurs irrespective of γ-secretase activity.

To map the domain(s) required for TREM2/PS1 interaction, we co-transfected PS1 and glutathione S-transferase (GST)-tagged full-length or truncated TREM2 into HEK293 cells and assayed for interactions between PS1 and TREM2-GST constructs in Glutathione Sepharose precipitates (Figures 1f and g). We found that TREM2 constructs comprising the transmembrane region (175–195 amino acids) co-precipitated with PS1, whereas TREM2 fragments lacking the transmembrane region pulled down much less PS1 (Figure 1g), suggesting that the TREM2 transmembrane domain is crucial for TREM2/PS1 interaction.

**Figure 2:**
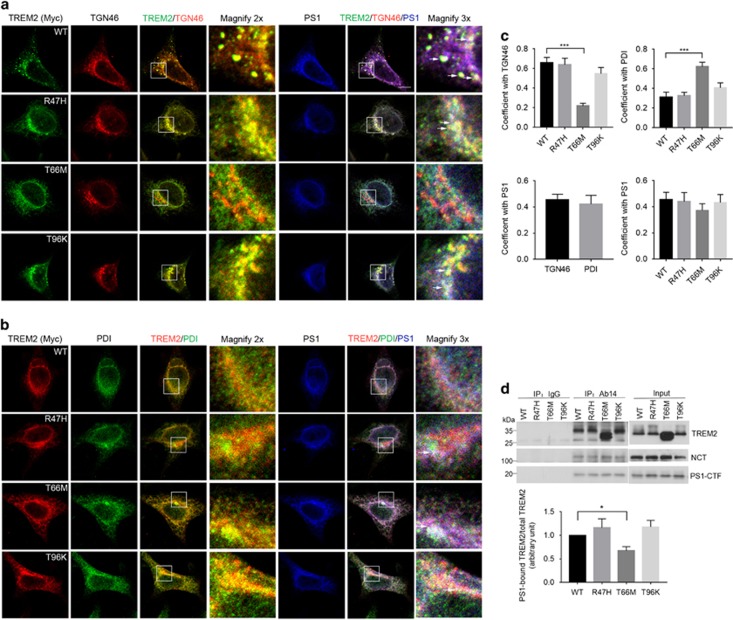
Mutations in TREM2 affect colocalization and interactions between TREM2 and presenilin 1 (PS1). PS1 was transfected into HEK293 cells stably expressing Myc-tagged TREM2 WT or TREM2 mutants as indicated. (**a**, **b**) Cells were then subjected to immunostaining with antibodies against Myc, PS1, and TGN46 (a marker for the Golgi, **a**) or PDI (a marker for the ER, **b**). White arrows in magnified images indicate colocalizing overlap for TREM2, PS1 and TGN46/PDI. Scale bars for **a**, **b**, 10 μm. (**c**) Quantification of colocalized signals. Pearson’s correlation coefficient is shown. ***P<0.001, *n*=3 independent experiments, one-way ANOVA with Dunnett’s *post hoc* analysis. (**d**) Cell lysates were immunoprecipitated with the PS1 antibody Ab14 or normal IgG. TREM2, NCT and PS1-CTF were detected by immunoblotting. The levels of precipitated TREM2 WT and mutants were normalized to the input. *P<0.05, *n*=3, Student’s *t*-test.

**Figure 3:**
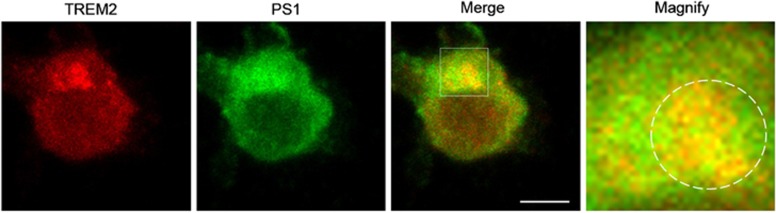
Endogenous TREM2 partially colocalizes with endogenous presenilin 1 (PS1) in microglial BV2 cells. BV2 cells were immunostained with antibodies against PS1 and mouse TREM2. White circles in magnified images indicate some colocalizing overlap between TREM2 and PS1 in Golgi-like structures. Scale bar, 5 μm.

Given that several disease-associated mutations in TREM2 have been previously described to alter its intracellular localization,^31, 32^ we generated HEK293 cell lines stably overexpressing TREM2 R47H, T66M and T96K mutants. We found that WT, R47H and T96K TREM2 predominantly localized in the Golgi apparatus, as seen by high colocalization coefficients observed with the Golgi marker TGN46, (Figures 2a and c), whereas T66M TREM2 primarily localized in the endoplasmic reticulum (ER) as indicated by colocalization with the ER marker PDI (Figures 2b and c). In addition, we observed that T66M mutation markedly reduced cell surface TREM2 expression, whereas R47H and T96K mutations did not affect surface expression of TREM2 (Supplementary Figure 1). PS1 could also be found in TGN46- or PDI-labeled structures (Figures 2a–c). Partial TREM2/PS1 colocalization was observed with the various TREM2 variants: specifically, staining overlap (white color) was observed among WT, R47H or T96K TREM2, PS1 and TGN46, and overlapping staining of T66M TREM2, PS1 and PDI was also apparent (Figures 2a–c). Although mutations in TREM2 did not influence PS1/TREM2 colocalization (Figure 2c), comparatively weaker T66M TREM2 interaction with PS1 was observed relative to the interaction between WT TREM2 and PS1, based on co-IP assays using the PS1 antibody Ab14 (Figure 2d). Moreover, we also observed colocalization between endogenous TREM2 and PS1 in the BV2 microglial cell line (Pearson’s colocalization coefficient, 0.41±0.11), primarily in Golgi-like compartments (Figure 3).

**Figure 4:**
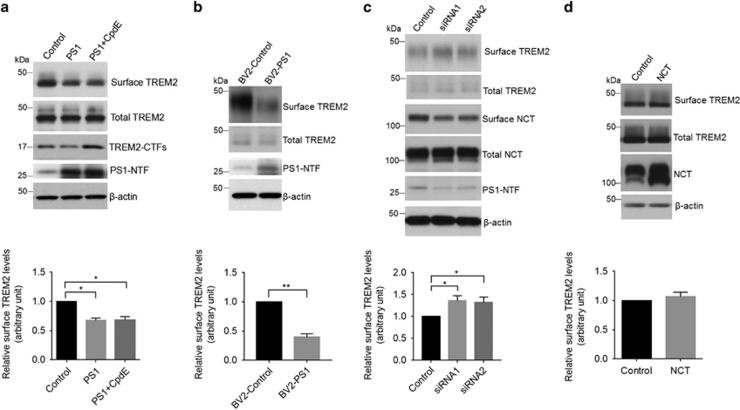
Upregulation of presenilin 1 (PS1) reduces steady-state levels of TREM2 at the cell surface. (**a**) Following PS1 overexpression, HEK293-TREM2 cells were treated with or without the γ-secretase inhibitor Compound E (CpdE) and subjected to cell surface biotinylation assay. Precipitates from streptavidin-agarose beads were immunoblotted for biotinylated TREM2 and total TREM2 levels (levels of TREM2 in 2% total cell lysates). *P<0.05, *n*=3, one-way ANOVA with Sidak *post hoc* analysis. (**b**) The level of endogenous TREM2 at the surface of microglial BV2 cells stably expressing PS1 (BV2-PS1) was determined by biotinylation. **P<0.01, *n*=3, Student’s *t*-test. (**c**) BV2 cells were transfected with a scrambled control siRNA or PS1-targeting siRNAs for 72 h. The level of cell surface TREM2 was determined by surface biotinylation. *P<0.05, *n*=3, Student’s *t*-test. (**d**) Cell surface expression of TREM2 in HEK293-TREM2 cells with NCT overexpression, as determined by biotinylation. *n*=3, Student’s *t*-test.

PS1 has been reported to regulate cell surface delivery of several transmembrane proteins such as amyloid precursor protein.^43, 44^ We next determined whether PS1 can affect surface expression of TREM2 by cell surface biotinylation assays. PS1 overexpression in HEK293-TREM2 cells resulted in a significant reduction in cell surface TREM2 levels with no change in total TREM2 levels (Figure 4a). Furthermore, we found that PS1 overexpression reduced cell surface TREM2 levels in the presence of the γ-secretase inhibitor CpdE, indicating that PS1-dependent depletion of TREM2 at the cell surface occurs independently of γ-secretase activity (Figure 4a). To confirm these effects in a microglia-derived cell line, we generated a murine microglial BV2 cell line stably overexpressing PS1, transduced with a lentiviral delivery system (BV2-PS1). We observed a reduction in endogenous TREM2 at the cell surface in BV2-PS1 cells compared with control BV2 cells (Figure 4b). To further clarify the regulatory effects of PS1 on TREM2 trafficking, we examined the effect of PS1 downregulation on cell surface TREM2 expression in BV2 cells. We found that PS1 knockdown significantly increased cell surface TREM2 levels (Figure 4c). As expected, cell surface NCT levels were concomitantly reduced upon PS1 knockdown (Figure 4c). We then investigated whether upregulation of other γ-secretase components such as NCT may have similar effects, and we found that overexpression of NCT had little effect on cell surface TREM2 distribution (Figure 4d). Together, these results indicate that TREM2 cell surface distribution is regulated by PS1 in a manner independent of the γ-secretase complex.

**Figure 5:**
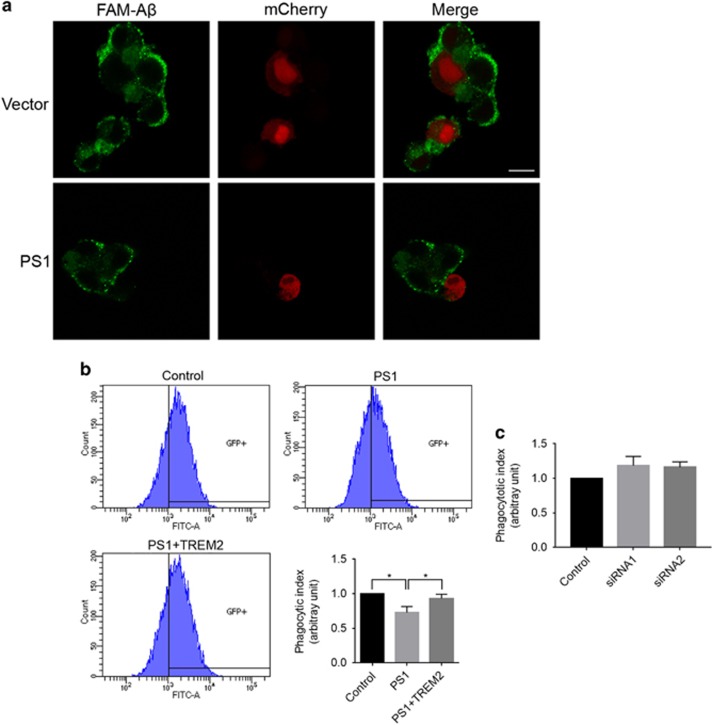
Overexpression of presenilin 1 (PS1) impairs TREM2-mediated phagocytosis in microglial cells. (**a**) PS1 and mCherry were co-transfected into BV2 microglial cells. The cells were then incubated with 6-carboxyfluorescein (FAM)-labeled Aβ42 for 2 h. FAM-Aβ42 uptake was analyzed by fluorescence microscopy. Scale bars, 10 μm. (**b**) Phagocytosis of FAM-Aβ42 in BV2 cells stably expressing PS1 as determined by flow cytometry. *P<0.05, *n*=3, one-way ANOVA with Sidak *post hoc* analysis. (**c**) Phagocytosis of FAM-Aβ42 in BV2 cells following PS1 knockdown as determined by flow cytometry. *n*=3, Student’s *t*-test.

The presence of TREM2 at the cell surface is important for transducing extracellular ligand signals to enact a microglial response. Several studies have reported that reduction of cell surface TREM2 expression in microglia cells can attenuate phagocytic function.^23, 31^ In support of this notion, transient overexpression of PS1 reduced phagocytic uptake of FAM-labeled Aβ42 within a 2-h incubation period in BV2 cells (Figure 5a). We further confirmed a reduction in FAM-Aβ42 uptake in BV2-PS1 cells by flow cytometry analysis (Figure 5b). To determine whether this reduction was TREM2-dependent, we transduced BV2-PS1 cells with lentiviruses expressing human TREM2. Strikingly, BV2-PS1 cells overexpressing TREM2 (BV2-PS1+TREM2) exhibited comparable phagocytic activity to control BV2 cells lacking PS1 and TREM2 overexpression (Figure 5b). In addition, we found that cell surface expression of TREM2 in BV2-PS1+TREM2 cells is comparable to that in BV2-control cells (Supplementary Figure 2). We observed no significant difference between the control and PS1 knockdown samples in bulk microglial phagocytic uptake (Figure 5c). Together, these results indicate that PS1 and TREM2 interact to reduce cell surface TREM2 distribution and downstream phagocytotic function.

## Discussion

TREM2 has been previously reported to be a substrate of the γ-secretase complex,^31, 33^ which suggests that TREM2 proteolysis could influence cell surface TREM2 distribution and function. Coordination of γ-secretase subunits including PSs, APH-1, NCT and PEN-2 is a prerequisite for γ-secretase-mediated proteolysis.^41^ Since total full-length TREM2 levels were unaffected by PS1 overexpression, this suggests that PS1 upregulation alone cannot enhance TREM2 proteolysis. Although inhibition of γ-secretase activity has recently been shown to reduce TREM2-mediated phagocytosis, which may be due to competitive binding between accumulated TREM2-CTF and DAP12, thereby impairing interactions between full-length TREM2 and DAP12 that are required for phagocytosis,^52^ our results demonstrate that PS1-dependent TREM2 trafficking remains unaffected by γ-secretase inhibition. Therefore, the effects of PS1 overexpression on TREM2 trafficking and microglial phagocytosis are likely not due to γ-secretase-dependent TREM2 cleavage in our system. Moreover, PS1 knockdown also had no effect on microglial phagocytosis. We speculate that cell surface full-length TREM2 and TREM2-CTF are both increased with PS1 knockdown, whereby both full-length TREM2 and TREM2-CTF can compete for DAP12 binding, thus producing no net effect on DAP12 signaling. On the whole, it is likely that PS1 can regulate the phagocytic function of TREM2 through both γ-secretase-dependent proteolytic and γ-secretase-independent trafficking roles.

Since TREM2 can be cleaved by (ADAM10A disintegrin and metalloproteinase domain-containing protein 10) on the cell surface,^31^ we speculate that overexpression of PS1 may reduce TREM2 ectodomain shedding. Although we do not know at this time how PS1 regulates TREM2 intracellular trafficking, several possibilities exist: one possibility is that PS1 affects the TREM2 trafficking from the Golgi/TGN; alternatively, PS1 may affect TREM2 internalization from the cell surface; a third possibility is that PS1 modulates the recycling of TREM2 from the endosomes and TGN to the cell surface. Future study may yield further information regarding how TREM2/PS1 interplay can affect consequent TREM2 distribution.

Various mutations identified in the TREM2 ectodomain exert differing effects on TREM2 structure. While R47H and T96K do not drastically affect TREM2 folding, T66M results in misfolding of TREM2 protein.^53^ Indeed, this may explain the altered subcellular localization of T66M TREM2 and reduced T66M/PS1 interaction that we observed. In addition to its role in mediating proteolytic catalysis within the γ-secretase complex, PS1 has been shown to execute other functions including the regulation of calcium homeostasis,^45, 46^ autophagy^47, 48^ and trafficking of various membrane proteins such as amyloid precursor protein.^43, 44^ Our results here point to a novel role for PS1 in regulating microglial phagocytosis through the regulation of TREM2 trafficking. Given that TREM2 signaling is also involved in the production of inflammatory cytokines,^25, 26, 28^ it may be of interest to further determine whether PS1 has a γ-secretase-independent role in mediating neuroinflammation. As neuroinflammatory processes such as microglial activation and cytokine generation are commonplace in multiple neurodegenerative diseases such as AD,^22, 54^ modulation of PS1/TREM2 trafficking may present alternative targeting strategies to treat AD. Since the use of γ-secretase inhibitors to inhibit Aβ generation has largely failed clinically,^55^ alternative strategies such as targeting PS1/TREM2 trafficking may be more effective in reversing cognitive decline near the onset of AD.

## Publisher’s note

Springer Nature remains neutral with regard to jurisdictional claims in published maps and institutional affiliations.

## References

[bib1] Guerreiro RJ, Lohmann E, Bras JM, Gibbs JR, Rohrer JD, Gurunlian N et al. Using exome sequencing to reveal mutations in TREM2 presenting as a frontotemporal dementia-like syndrome without bone involvement. JAMA Neurol 2013; 70: 78–84. 23318515 10.1001/jamaneurol.2013.579 PMC4001789

[bib2] Giraldo M, Lopera F, Siniard AL, Corneveaux JJ, Schrauwen I, Carvajal J et al. Variants in triggering receptor expressed on myeloid cells 2 are associated with both behavioral variant frontotemporal lobar degeneration and Alzheimer's disease. Neurobiol Aging 2013; 34: 2077 e11–2077 e18. 10.1016/j.neurobiolaging.2013.02.016 PMC3830921 23582655

[bib3] Rayaprolu S, Mullen B, Baker M, Lynch T, Finger E, Seeley WW et al. TREM2 in neurodegeneration: evidence for association of the p.R47H variant with frontotemporal dementia and Parkinson's disease. Mol Neurodegener 2013; 8: 19. 23800361 10.1186/1750-1326-8-19 PMC3691612

[bib4] Le Ber I, De Septenville A, Guerreiro R, Bras J, Camuzat A, Caroppo P et al. Homozygous TREM2 mutation in a family with atypical frontotemporal dementia. Neurobiol Aging 2014; 35: 2419.e23–2419.e25. 10.1016/j.neurobiolaging.2014.04.010 PMC4208293 24910390

[bib5] Borroni B, Ferrari F, Galimberti D, Nacmias B, Barone C, Bagnoli S et al. Heterozygous TREM2 mutations in frontotemporal dementia. Neurobiol Aging 2014; 35: 934 e7–934.e10. 10.1016/j.neurobiolaging.2013.09.017 24139279

[bib6] Cuyvers E, Bettens K, Philtjens S, Van Langenhove T, Gijselinck I, van der Zee J et al. Investigating the role of rare heterozygous TREM2 variants in Alzheimer’s disease and frontotemporal dementia. Neurobiol Aging 2014; 35: 726 e11–726 e19. 10.1016/j.neurobiolaging.2013.09.009 24119542

[bib7] Cady J, Koval ED, Benitez BA, Zaidman C, Jockel-Balsarotti J, Allred P et al. TREM2 variant p.R47H as a risk factor for sporadic amyotrophic lateral sclerosis. JAMA Neurol 2014; 71: 449–453. 24535663 10.1001/jamaneurol.2013.6237 PMC4087113

[bib8] Guerreiro R, Wojtas A, Bras J, Carrasquillo M, Rogaeva E, Majounie E et al. TREM2 variants in Alzheimer’s disease. N Engl J Med 2013; 368: 117–127. 23150934 10.1056/NEJMoa1211851 PMC3631573

[bib9] Jonsson T, Stefansson H, Steinberg S, Jonsdottir I, Jonsson PV, Snaedal J et al. Variant of TREM2 associated with the risk of Alzheimer’s disease. N Engl J Med 2013; 368: 107–116. 23150908 10.1056/NEJMoa1211103 PMC3677583

[bib10] Benitez BA, Cooper B, Pastor P, Jin SC, Lorenzo E, Cervantes S et al. TREM2 is associated with the risk of Alzheimer’s disease in Spanish population. Neurobiol Aging 2013; 34: 1711 e15–1711 e17. 10.1016/j.neurobiolaging.2012.12.018 PMC3596468 23391427

[bib11] Jin SC, Carrasquillo MM, Benitez BA, Skorupa T, Carrell D, Patel D et al. TREM2 is associated with increased risk for Alzheimer's disease in African Americans. Mol Neurodegener 2015; 10: 19. 25886450 10.1186/s13024-015-0016-9 PMC4426167

[bib12] Korvatska O, Leverenz JB, Jayadev S, McMillan P, Kurtz I, Guo X et al. R47H variant of TREM2 associated with Alzheimer disease in a large late-onset family: clinical, genetic, and neuropathological study. JAMA Neurol 2015; 72: 920–927. 26076170 10.1001/jamaneurol.2015.0979 PMC4825672

[bib13] Neumann H, Daly MJ. Variant TREM2 as risk factor for Alzheimer’s disease. N Engl J Med 2013; 368: 182–184. 23151315 10.1056/NEJMe1213157

[bib14] Atagi Y, Liu CC, Painter MM, Chen XF, Verbeeck C, Zheng H et al. Apolipoprotein E is a ligand for triggering receptor expressed on myeloid cells 2 (TREM2). J Biol Chem 2015; 290: 26043–26050. 26374899 10.1074/jbc.M115.679043 PMC4646257

[bib15] Bailey CC, DeVaux LB, Farzan M. The triggering receptor expressed on myeloid cells 2 binds apolipoprotein E. J Biol Chem 2015; 290: 26033–26042. 26374897 10.1074/jbc.M115.677286 PMC4646256

[bib16] Yeh FL, Wang Y, Tom I, Gonzalez LC, Sheng M. TREM2 binds to apolipoproteins, including APOE and CLU/APOJ, and thereby facilitates uptake of amyloid-beta by microglia. Neuron 2016; 91: 328–340. 27477018 10.1016/j.neuron.2016.06.015

[bib17] Bouchon A, Hernandez-Munain C, Cella M, Colonna M. A DAP12-mediated pathway regulates expression of CC chemokine receptor 7 and maturation of human dendritic cells. J Exp Med 2001; 194: 1111–1122. 11602640 10.1084/jem.194.8.1111 PMC2193511

[bib18] Sessa G, Podini P, Mariani M, Meroni A, Spreafico R, Sinigaglia F et al. Distribution and signaling of TREM2/DAP12, the receptor system mutated in human polycystic lipomembraneous osteodysplasia with sclerosing leukoencephalopathy dementia. Eur J Neurosci 2004; 20: 2617–2628. 15548205 10.1111/j.1460-9568.2004.03729.x

[bib19] Schmid CD, Sautkulis LN, Danielson PE, Cooper J, Hasel KW, Hilbush BS et al. Heterogeneous expression of the triggering receptor expressed on myeloid cells-2 on adult murine microglia. J Neurochem 2002; 83: 1309–1320. 12472885 10.1046/j.1471-4159.2002.01243.x PMC2637869

[bib20] Colonna M. TREMs in the immune system and beyond. Nat Rev Immunol 2003; 3: 445–453. 12776204 10.1038/nri1106

[bib21] Forabosco P, Ramasamy A, Trabzuni D, Walker R, Smith C, Bras J et al. Insights into TREM2 biology by network analysis of human brain gene expression data. Neurobiol Aging 2013; 34: 2699–2714. 23855984 10.1016/j.neurobiolaging.2013.05.001 PMC3988951

[bib22] Painter MM, Atagi Y, Liu CC, Rademakers R, Xu H, Fryer JD et al. TREM2 in CNS homeostasis and neurodegenerative disease. Mol Neurodegener 2015; 10: 43. 26337043 10.1186/s13024-015-0040-9 PMC4560063

[bib23] Takahashi K, Rochford CD, Neumann H. Clearance of apoptotic neurons without inflammation by microglial triggering receptor expressed on myeloid cells-2. J Exp Med 2005; 201: 647–657. 15728241 10.1084/jem.20041611 PMC2213053

[bib24] Hsieh CL, Koike M, Spusta SC, Niemi EC, Yenari M, Nakamura MC et al. A role for TREM2 ligands in the phagocytosis of apoptotic neuronal cells by microglia. J Neurochem 2009; 109: 1144–1156. 19302484 10.1111/j.1471-4159.2009.06042.x PMC3087597

[bib25] Wang Y, Cella M, Mallinson K, Ulrich JD, Young KL, Robinette ML et al. TREM2 lipid sensing sustains the microglial response in an Alzheimer’s disease model. Cell 2015; 160: 1061–1071. 25728668 10.1016/j.cell.2015.01.049 PMC4477963

[bib26] Zhong L, Chen XF, Zhang ZL, Wang Z, Shi XZ, Xu K et al. DAP12 stabilizes the C-terminal fragment of the triggering receptor expressed on myeloid cells-2 (TREM2) and protects against LPS-induced pro-inflammatory response. J Biol Chem 2015; 290: 15866–15877. 25957402 10.1074/jbc.M115.645986 PMC4505493

[bib27] Kawabori M, Kacimi R, Kauppinen T, Calosing C, Kim JY, Hsieh CL et al. Triggering receptor expressed on myeloid cells 2 (TREM2) deficiency attenuates phagocytic activities of microglia and exacerbates ischemic damage in experimental stroke. J Neurosci 2015; 35: 3384–3396. 25716838 10.1523/JNEUROSCI.2620-14.2015 PMC4339351

[bib28] Zheng H, Liu CC, Atagi Y, Chen XF, Jia L, Yang L et al. Opposing roles of the triggering receptor expressed on myeloid cells 2 and triggering receptor expressed on myeloid cells-like transcript 2 in microglia activation. Neurobiol Aging 2016; 42: 132–141. 27143430 10.1016/j.neurobiolaging.2016.03.004 PMC4857884

[bib29] Malik M, Parikh I, Vasquez JB, Smith C, Tai L, Bu G et al. Genetics ignite focus on microglial inflammation in Alzheimer’s disease. Mol Neurodegener 2015; 10: 52. 26438529 10.1186/s13024-015-0048-1 PMC4595327

[bib30] Heslegrave A, Heywood W, Paterson R, Magdalinou N, Svensson J, Johansson P et al. Increased cerebrospinal fluid soluble TREM2 concentration in Alzheimer’s disease. Mol Neurodegener 2016; 11: 3. 26754172 10.1186/s13024-016-0071-x PMC4709982

[bib31] Kleinberger G, Yamanishi Y, Suarez-Calvet M, Czirr E, Lohmann E, Cuyvers E et al. TREM2 mutations implicated in neurodegeneration impair cell surface transport and phagocytosis. Sci Transl Med 2014; 6: 243ra86. 10.1126/scitranslmed.3009093 24990881

[bib32] Park JS, Ji IJ, An HJ, Kang MJ, Kang SW, Kim DH et al. Disease-associated mutations of TREM2 alter the processing of N-linked oligosaccharides in the Golgi apparatus. Traffic 2015; 16: 510–518. 25615530 10.1111/tra.12264

[bib33] Wunderlich P, Glebov K, Kemmerling N, Tien NT, Neumann H, Walter J. Sequential proteolytic processing of the triggering receptor expressed on myeloid cells-2 (TREM2) protein by ectodomain shedding and gamma-secretase-dependent intramembranous cleavage. J Biol Chem 2013; 288: 33027–33036. 24078628 10.1074/jbc.M113.517540 PMC3829152

[bib34] Glenner GG, Wong CW. Alzheimer's disease: initial report of the purification and characterization of a novel cerebrovascular amyloid protein. Biochem Biophys Res Commun 1984; 120: 885–890. 6375662 10.1016/s0006-291x(84)80190-4

[bib35] Tu S, Okamoto S, Lipton SA, Xu H. Oligomeric Aβ-induced synaptic dysfunction in Alzheimer’s disease. Mol Neurodegener 2014; 9: 48. 25394486 10.1186/1750-1326-9-48 PMC4237769

[bib36] Selkoe DJ, Hardy J. The amyloid hypothesis of Alzheimer’s disease at 25 years. EMBO Mol Med 2016; 8: 595–608. 27025652 10.15252/emmm.201606210 PMC4888851

[bib37] Doens D, Fernandez PL. Microglia receptors and their implications in the response to amyloid beta for Alzheimer’s disease pathogenesis. J Neuroinflammation 2014; 11: 48. 24625061 10.1186/1742-2094-11-48 PMC3975152

[bib38] Kimberly WT, LaVoie MJ, Ostaszewski BL, Ye W, Wolfe MS, Selkoe DJ. Gamma-secretase is a membrane protein complex comprised of presenilin, nicastrin, Aph-1, and Pen-2. Proc Natl Acad Sci USA 2003; 100: 6382–6387. 12740439 10.1073/pnas.1037392100 PMC164455

[bib39] De Strooper B. Aph-1, Pen-2, and Nicastrin with Presenilin generate an active gamma-Secretase complex. Neuron 2003; 38: 9–12. 12691659 10.1016/s0896-6273(03)00205-8

[bib40] Thinakaran G, Borchelt DR, Lee MK, Slunt HH, Spitzer L, Kim G et al. Endoproteolysis of presenilin 1 and accumulation of processed derivatives *in vivo*. Neuron 1996; 17: 181–190. 8755489 10.1016/s0896-6273(00)80291-3

[bib41] Zhang X, Li Y, Xu H, Zhang YW. The gamma-secretase complex: from structure to function. Front Cell Neurosci 2014; 8: 427. 25565961 10.3389/fncel.2014.00427 PMC4263104

[bib42] Vetrivel KS, Zhang YW, Xu H, Thinakaran G. Pathological and physiological functions of presenilins. Mol Neurodegener 2006; 1: 4. 16930451 10.1186/1750-1326-1-4 PMC1513131

[bib43] Cai D, Leem JY, Greenfield JP, Wang P, Kim BS, Wang R et al. Presenilin-1 regulates intracellular trafficking and cell surface delivery of beta-amyloid precursor protein. J Biol Chem 2003; 278: 3446–3454. 12435726 10.1074/jbc.M209065200

[bib44] Naruse S, Thinakaran G, Luo JJ, Kusiak JW, Tomita T, Iwatsubo T et al. Effects of PS1 deficiency on membrane protein trafficking in neurons. Neuron 1998; 21: 1213–1221. 9856475 10.1016/s0896-6273(00)80637-6

[bib45] Zhang H, Sun S, Herreman A, De Strooper B, Bezprozvanny I. Role of presenilins in neuronal calcium homeostasis. J Neurosci 2010; 30: 8566–8580. 20573903 10.1523/JNEUROSCI.1554-10.2010 PMC2906098

[bib46] Green KN, Demuro A, Akbari Y, Hitt BD, Smith IF, Parker I et al. SERCA pump activity is physiologically regulated by presenilin and regulates amyloid beta production. J Cell Biol 2008; 181: 1107–1116. 18591429 10.1083/jcb.200706171 PMC2442205

[bib47] Lee JH, Yu WH, Kumar A, Lee S, Mohan PS, Peterhoff CM et al. Lysosomal proteolysis and autophagy require presenilin 1 and are disrupted by Alzheimer-related PS1 mutations. Cell 2010; 141: 1146–1158. 20541250 10.1016/j.cell.2010.05.008 PMC3647462

[bib48] Zhang X, Garbett K, Veeraraghavalu K, Wilburn B, Gilmore R, Mirnics K et al. A role for presenilins in autophagy revisited: normal acidification of lysosomes in cells lacking PSEN1 and PSEN2. J Neurosci 2012; 32: 8633–8648. 22723704 10.1523/JNEUROSCI.0556-12.2012 PMC3467018

[bib49] Duggan SP, McCarthy JV. Beyond gamma-secretase activity: The multifunctional nature of presenilins in cell signalling pathways. Cell Signal 2016; 28: 1–11. 10.1016/j.cellsig.2015.10.006 26498858

[bib50] Zhao Y, Tseng IC, Heyser CJ, Rockenstein E, Mante M, Adame A et al. Appoptosin-mediated caspase cleavage of tau contributes to progressive supranuclear palsy pathogenesis. Neuron 2015; 87: 963–975. 26335643 10.1016/j.neuron.2015.08.020 PMC4575284

[bib51] Wolfe MS, Xia W, Ostaszewski BL, Diehl TS, Kimberly WT, Selkoe DJ. Two transmembrane aspartates in presenilin-1 required for presenilin endoproteolysis and gamma-secretase activity. Nature 1999; 398: 513–517. 10206644 10.1038/19077

[bib52] Glebov K, Wunderlich P, Karaca I, Walter J. Functional involvement of gamma-secretase in signaling of the triggering receptor expressed on myeloid cells-2 (TREM2). J Neuroinflammation 2016; 13: 17. 26792193 10.1186/s12974-016-0479-9 PMC4721188

[bib53] Kober DL, Alexander-Brett JM, Karch CM, Cruchaga C, Colonna M, Holtzman MJ et al. Neurodegenerative disease mutations in TREM2 reveal a functional surface and distinct loss-of-function mechanisms. Elife 2016; 20: 5. 10.7554/eLife.20391 PMC5173322 27995897

[bib54] Glass CK, Saijo K, Winner B, Marchetto MC, Gage FH. Mechanisms underlying inflammation in neurodegeneration. Cell 2010; 140: 918–934. 20303880 10.1016/j.cell.2010.02.016 PMC2873093

[bib55] De Strooper B, Chavez Gutierrez L. Learning by failing: ideas and concepts to tackle gamma-secretases in Alzheimer's disease and beyond. Annu Rev Pharmacol Toxicol 2015; 55: 419–437. 25292430 10.1146/annurev-pharmtox-010814-124309

