# Supplementary material for: Intracellular trafficking of TREM2 is regulated by presenilin 1
Source: Exp Mol Med. 2017 Dec 1;49(12):e405–. doi: 10.1038/emm.2017.200 (PMC5750471; doi:10.1038/emm.2017.200)
Supplement: Supplementary Information [file emm2017200x1.docx]

**Supplementary Figures**


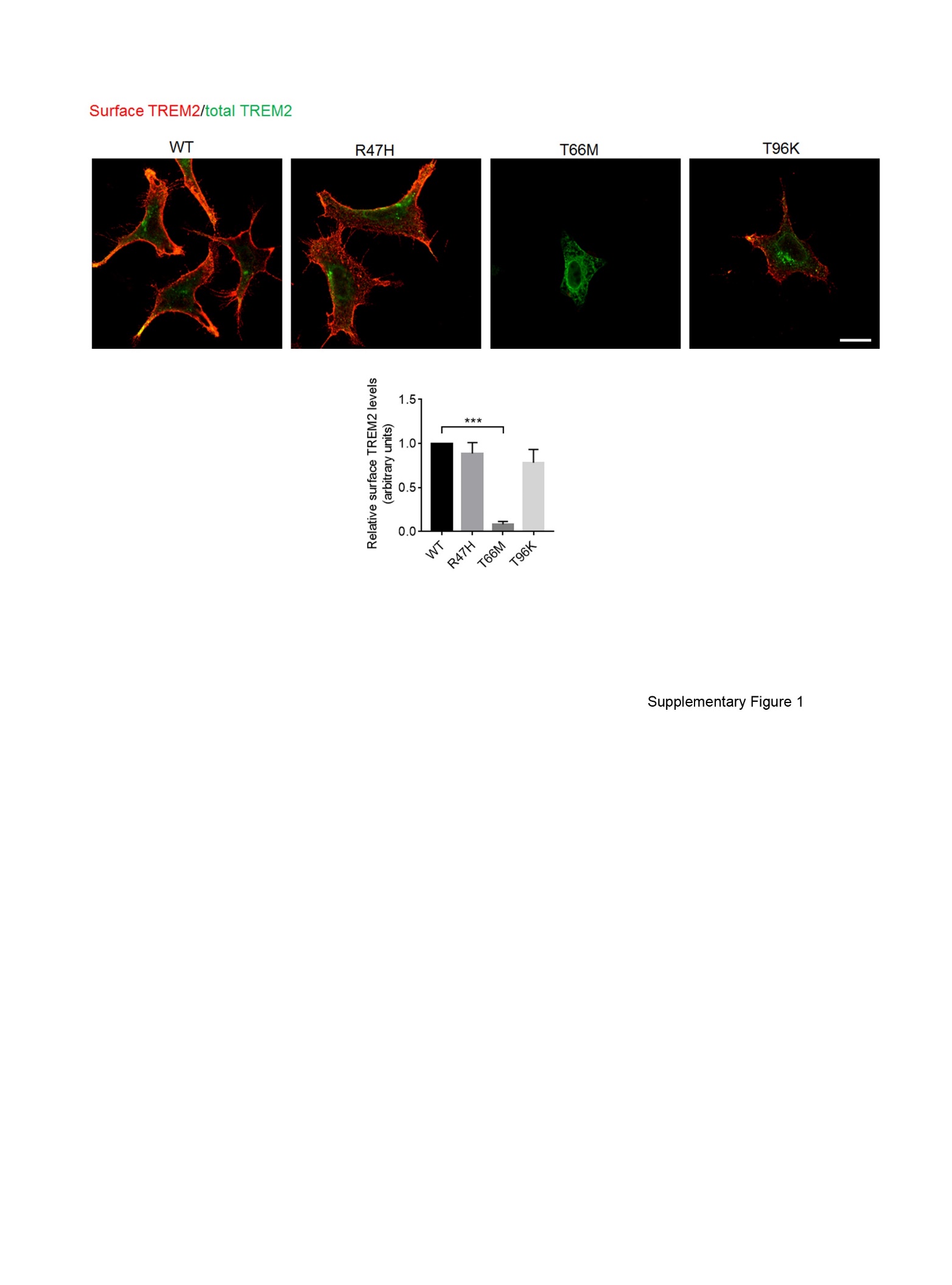


**Supplementary Figure 1** The T66M mutation reduces cell surface TREM2 expression. Cell surface TREM2 (red) was labeled with an antibody against the TREM2 N-terminus (19-174) under non-permeabilization conditions, whereas total TREM2 (green) was stained with a Myc antibody after permeabilization. Scale bar, 10 µm. ***, *p* < 0.001, *n*=3, one-way ANOVA with Dunnett’s post hoc analysis.


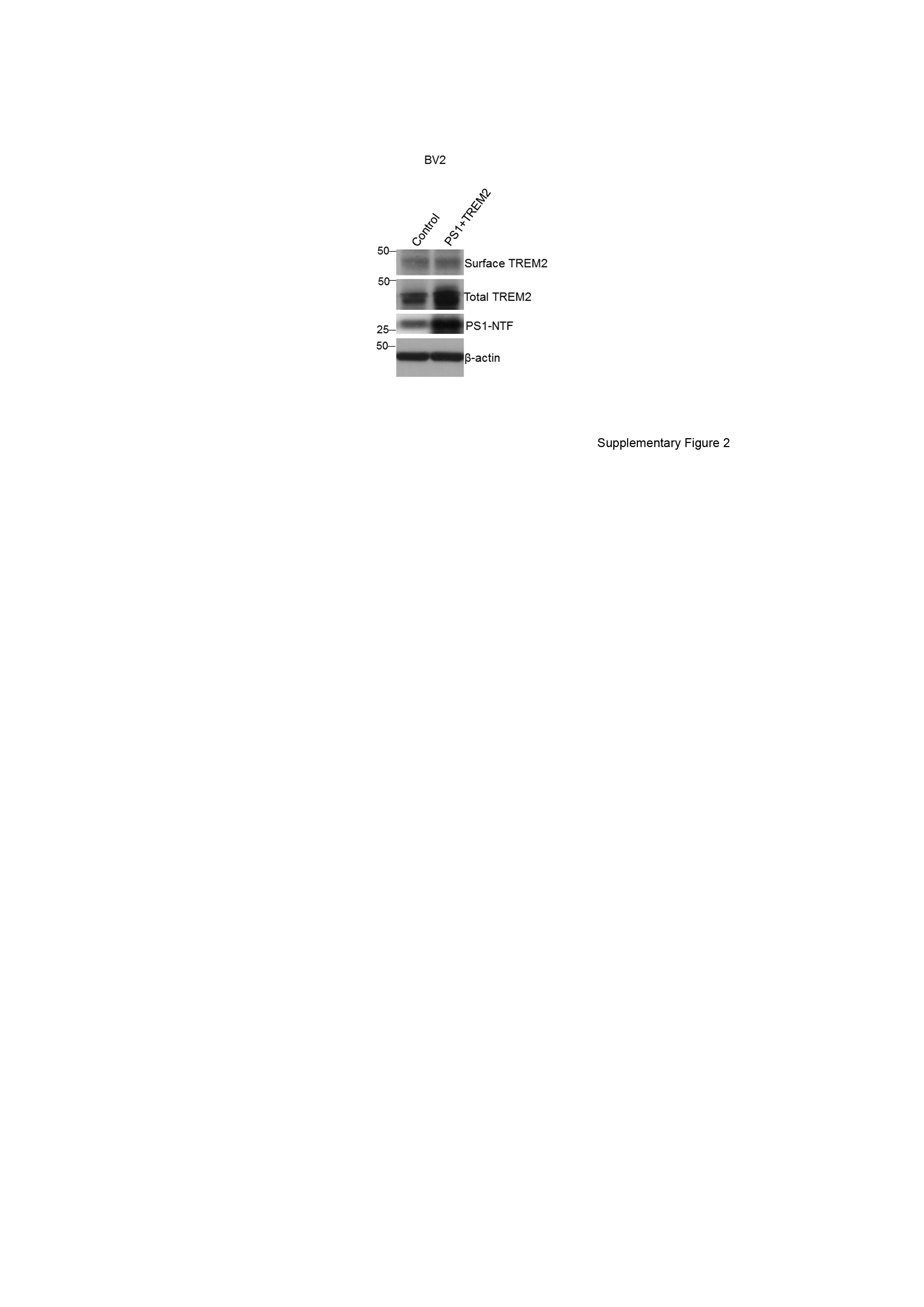


**Supplementary Figure 2** Cell surface TREM2 expression is unaltered in BV2-PS1+TREM2 cells. BV2-control or BV2-PS1+TREM2 cells were subjected to cell surface biotinylation labeling. Biotinylated and total TREM2 as well as PS1-NTF and β-actin were detected by immunoblotting.
